# Animal-Protein-Based and Synthetic-Based Foamed Mixture Lightweight Soil Doped with Bauxite Tailings: Macro and Microscopic Properties

**DOI:** 10.3390/ma15186377

**Published:** 2022-09-14

**Authors:** Xiaoduo Ou, Peng Mo, Zhengfan Lyu, Junhui Luo, Jie Jiang, Lu Bai, Zhongzheng Huang

**Affiliations:** 1 School of Civil Engineering and Architecture, Guangxi University, Nanning 530004, China; 2 Guangxi Beitou Transportation Maintenance Technology Group Co., Ltd., Nanning 530029, China; 3 Guangxi Traffic Construction Engineering Testing Consulting Co., Ltd., Nanning 530012, China; 4 Guangxi Engineering Research Center for Comprehensive Utilization of Bauxite Tailings, Nanning 530004, China; 5 Guangxi Ruiyu Construction Technology Co., Ltd., Nanning 530000, China

**Keywords:** foamed mixture lightweight soil mixed with BTs (FMLSB), foaming agent, compressive strength, fluidity, volume of water absorption

## Abstract

In order to explore the effect of the foaming agent type on the properties of foamed mixture lightweight soil mixed with bauxite tailings (FMLSB), low-density (437.5 kg/m^3^ and 670 kg/m^3^) and high-density (902.5 kg/m^3^ and 1170 kg/m^3^) FMLSB were prepared using protein-based and synthetic-based foaming agents (AF and SF, respectively). The foam stability, micro characteristics, compressive strength, fluidity, and volume of water absorption of the FMLSB were investigated. The results showed that the foam made from AF had better strength and stability compared to SF. The internal pore sizes of both AF- and SF-FMLSB at low density were large, but at high density the internal pore sizes and area porosity of AF-FMLSB were smaller than those of SF-FMLSB. In terms of compressive strength, the compressive strength of AF-FMLSB was improved by 17.5% to 43.2% compared to SF-FMLSB. At low density, the fluidity of AF- and SF-FMLSB is similar, while at high density the fluidity of AF-FMLSB is much higher than that of SF-FMLSB. In addition, the stable volume of water absorption of SF-FMLSB is smaller than that of AF-FMLSB at low density, and the corresponding water resistance is better, but the situation is reversed at high density.

## 1. Introduction

Foamed mixture lightweight soil (FMLS) is prepared by mixing cement slurry with foam generated by foaming agents. Because of its advantages of light weight, adjustable strength and density, and high economy [1,2,3], it has been widely used in the engineering fields of heat preservation and insulation, soft foundation treatment, pipeline landfill, and filling of mining areas [4,5,6]. Based on the excellent performance of FMLS, Ou et al. [6] and Peng et al. [7,8] carried out studies on the resource utilization of a large amount of high-water-content bauxite tailings (BTs), so as to fulfill the purpose of improving aluminum industrial land in western Guangxi and reducing the safety risk of old tailings ponds. They used bauxite tailings that were non-toxic [9], with fine particle size and high contents of Al_2_O_3_ and SiO_2_ [7], as raw materials to successfully prepare a kind of foamed mixture lightweight soil with BTs (FMLSB), with excellent performance and the ability to meet the requirements of cavity filling. However, the current research on FMLSB is still limited to its physical and mechanical properties.

As a porous material, the macroscopic properties of FMLS are affected by its internal pores [10,11], and since the internal pores are introduced by the foaming agent, different types of foaming agents also have an impact on the properties of the FMLS [12]. So far, many studies have been published on the relationship between foaming agents and FMLS performance. Huang et al. [13] analyzed the effects of different types of foaming agents on the physical and mechanical properties of FMLS, and the results showed that the FMLS prepared using animal-protein-based foaming agents had better properties. Falliano D. et al. [14] found that the foaming agent type has a significant effect on the compressive strength of medium–low-density FMLS, with a wet density ranging from 500 kg/m^3^ to 1000 kg/m^3^. Panesar D.K. [15] investigated the effects of foaming agent type on the properties of medium–high-density FMLS with foam volume fractions of 6% to 35%, coming to the conclusion that there was a significant effect of foaming agent type on thermal resistance and adsorption coefficient, but less on mechanical properties. Meanwhile, Kim J.M. et al. [16] concluded that the compressive strength and thermal conductivity of low-density FMLS using different foaming agent types were similar, while differences existed for high-density FMLS. Sun et al. [17] and Panesar D.K. [15] investigated the reasons for the effects of foaming agent type on the macroscopic properties of FMLS, and concluded that the foaming agent leads to differences in the physical and mechanical properties and durability performance of FMLS by affecting the pore characteristics and pore connectivity of FMLS. In addition, He et al. [18], Hashim M. et al. [19], and Li et al. [20] found that foam stability is an important factor affecting the pore structure of FMLS, i.e., the better the foam stability, the better the performance of the FMLS. The addition of nanoparticles (e.g., nanoalumina and nanosilica) and organic compounds (e.g., hydroxypropyl methylcellulose) to modify the foaming agent can also enhance the foam stability and, thus, improve the performance of FMLS [21,22,23]. From the above studies, it can be seen that there are different degrees of difference in the macroscopic properties of the FMLSs prepared with different foaming agents. The effect of the foaming agent on the performance of FMLSB, as a new material, remains unclear. Therefore, it is necessary to conduct a study on the effect of foaming agent type on the physical and mechanical properties of bauxite tailing sludge FMLS.

The current most commonly used foaming agents are mainly synthetic-based (SF), animal/plant-protein-based (AF and PF, respectively), and rosin-based (RF). The foaming multiples and foam stability of PF and RF are usually poor [24]. In this study, therefore, SF and AF were selected as foaming agents. Four wet densities of FMLSB were designed for this investigation. The stability of the two foaming agents was studied first, and then the effects of the two foaming agents on the compressive strength of the four wet densities of FMLSB were investigated based on the results of microscopic analysis. Considering the use of FMLSB as a filling material, the effects of two foaming agents on the fluidity and the volume of water absorption of FMLSB were also discussed. The results in this paper can provide a scientific basis for promoting the application of FMLSB in the field of filling.

## 2. Materials and Methods

### 2.1. Materials

**Figure 1:**
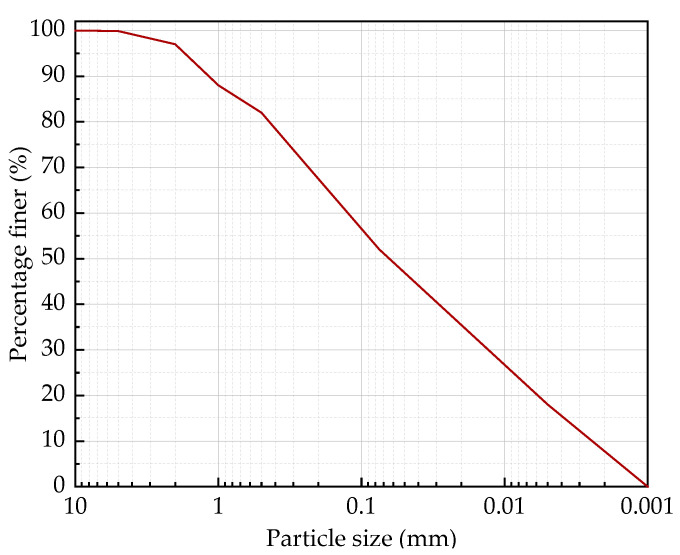
Particle size distribution curve of BTs.

Cement: Chinese Portland cement (P·O 42.5) with a density of 3.10 g/cm^3^ and a specific surface area of 341 m^2^/kg produced by Fusui Xinning conch cement company Nanning, China. BTs were taken from the central depression of the No.1 tailings pond of the Aluminum Corporation of China’s Limited Guangxi Branch. Its particle size curve, along with its main physical parameters and chemical composition, are presented in Figure 1 and Table 1, respectively. It can be seen that the BTs had a high fine particle content, and their main chemical composition was Al_2_O_3_, SiO_2_, and Fe_2_O_3_. An AF called FP-180 (trademark name) and an SF called PCFA (acronym standing for polymer composite foaming agent) were used as foaming agents, which were supplied by Ketai Energy-saving Building Material Co., Ltd., Linyi, China., and Baile Energy Equipment Co., Ltd., Hefei, China., respectively. According to the recommendations of the foaming agent manufacturer, the dilution ratios of SF and AF were 1:40 and 1:30 respectively. The performance parameters of SF and AF are shown in Table 2.

### 2.2. Mix Proportion

With reference to the relevant provisions of the “Technical specification for foamed mixture lightweight soil filling engineering” (CJJ/T177-2012) [25], four wet density grades were designed for this test, and the ratios of each raw material are shown in Table 3.

### 2.3. Specimen Preparation and Curing

**Figure 2:**
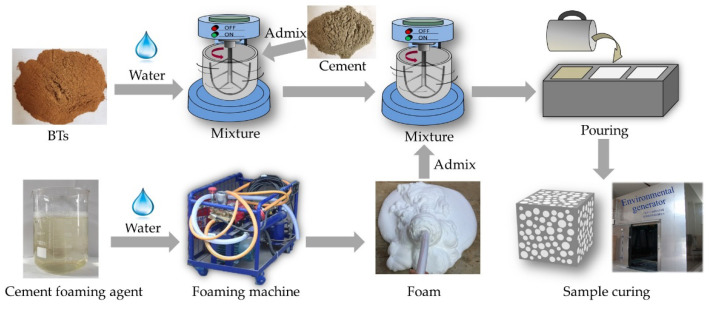
Schematic diagram of the FMLSB preparation process.

There are two methods to prepare FMLS: physical and chemical foaming methods. In this study, FMLSB was prepared via the physical foaming method; its preparation process shown in Figure 2. Firstly, we weighed the cement, BTs, and water according to the design ratio, and added them to the blender to mix evenly. Secondly, we diluted the foaming agent with water according to the best dilution ratio and delivered it to the foaming machine to make foam. The foaming machine was composed of an air compressor, a water pump, and a foaming cylinder. Its working principle is to use the compressed air generated by the foaming machine to transport the aqueous foaming agent solution and compressed air to the foaming cylinder (filled with steel wire balls). At the same time, the compressed air pushes the foaming agent solution through the steel ball to produce uniform and fine bubbles. Finally, according to the mix proportion, the corresponding volume of foam was transported to the blender and mixed evenly to make fresh FMLSB slurry, which was then poured into 10 × 10 × 10 cm^3^ molds. The molds with FMLSB slurry were covered with a plastic film and stored in the environmental generator [26] for curing at a relative humidity of 95 ± 3% and temperature of 22 ± 2 °C for 48 h. After removing the molds, hardened FMLSB specimens were sealed in valve bags and cured at a relative humidity of 95 ± 3% and temperature of 22 ± 2 °C for 28 d.

### 2.4. Test Methods

Referring to Chinese Standard CJJ/T 177–2012 [25], the fluidity of the fresh FMLSB slurry was tested with a cylindrical die (the height and internal diameter of the opening were 80 mm, and the inner wall was smooth). The prepared FMLSB was poured into the cylindrical die, and scraped flat with a scraper. Then, the cylindrical die was slowly lifted vertically. After standing still for 1 min, we measured the maximum diameter with a Vernier caliper. Repeated measurements were taken 3 times, and the arithmetic mean was taken as the fluidity of the FMLSB.

Foam stability test methods followed the method of Sun et al. [17]. The foams prepared with different foaming agents were collected in beaker (capacity 1000 mL, height 155 mm). Two balls with the same size, diameter, and weight were placed gently on the top of the foam. To accelerate the test process, the balls were filled with water until their weight reached 10 g. A camera was used to record the position of the small ball at 0 min, 3 min, and 5 min (after which it was recorded once every 5 min until it reached the bottom). This method can be more intuitive to understand the stability of the foam.

Unconfined compressive strength (UCS) was tested on a WAW-600 microcomputer-controlled electrohydraulic servo universal testing machine (produced by Shanghai Hualong Testing Instrument Co., Ltd., Shanghai, China). The displacement control method was used for loading, with a loading speed of 5 mm·min^−1^. The arithmetic mean value of the measured values of three FMLSB samples was taken as the unconfined compressive strength of the FMLSB.

Microanalysis: The central part of the FMLSB specimen was used as an SEM observation sample with dimensions of 4 mm × 8 mm × 4 mm, and its natural structural surface was used as the observation surface. Scanning electron microscope (SEM) images of the pore structure and skeletal structure of FMLSB samples were observed using an S-3400N (produced by Hitachi Limited, Tokyo, Japan). The observation magnification was 50× and 1000×, respectively. 

**Figure 3:**
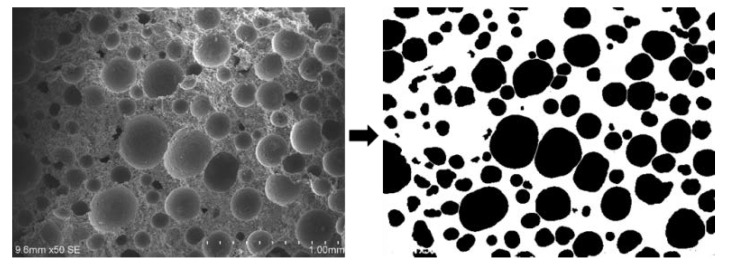
Binary processing of FMLSB pores.

Area porosity measurement: We binarized the obtained SEM images, and then calculated the area porosity of the FMLSB with Image-Pro Plus (IPP) software. The binarization process is shown in Figure 3 (the black parts represent the pores of the sample, while the white part is the skeleton of the sample).

Water absorption: Three samples from each group cured for 28 days were soaked in distilled water at 20 °C, and their weight was measured at 0, 1, 2, 3, 4, 5, 6, 8, 10, 15, 20, 25, 30, 40, 50, and 60 days. The following formula was used to calculate the volume of water absorption by the FMLSB. The arithmetic mean value of the measured values of three FMLSB samples was taken as the volume of water absorption by the FMLSB. According to the test data, the relationship between the volume of water absorption and the soaking time of bauxite tailings in foam lightweight soil can be obtained as follows:

(1) $$\mathrm{VA}=\frac{m_{i}{-m}_{0}}{\rho_{w}V_{0}}\times 100\%$$

where *VA* is the volume of water absorption by the FMLSB, *V*_0_ is the volume of the FMLSB (m^3^), *m*_i_ is the weight of the FMLSB samples immediately after the ith day of soaking (kg), *m*_0_ is the initial weight of FMLSB (kg), and *ρ*_w_ is the density of water, taken as 1000 kg·m^−3^.

## 3. Results and Discussions

### 3.1. Foaming Agent Performance Test

#### 3.1.1. Foaming Agent General Performance

According to Chinese Standard CJJ/T 177–2012 [25], the general performance indices of foaming agents are mainly 1 h water secretion and 1 h sink distance, and the performance indices of the two foaming agents measured in this test are shown in Table 2. It can be seen that the general performance indices of the AF and SF foaming agents were essentially the same at the optimal dilution, and only the water secretion differed somewhat. However, Li et al. [27] measured the 1 h water secretion and 1 h sink distance of nine kinds of homemade foaming agents and three kinds of commercially available foaming agents, and found that some foaming agents with a small sink distance had high water secretion instead. This indicates that the performance indicators of foaming agents in the current Chinese standard (e.g., water secretion, sink distance) cannot really reflect the performance differences of the foaming agents. 

#### 3.1.2. Foam Stability

**Figure 4:**
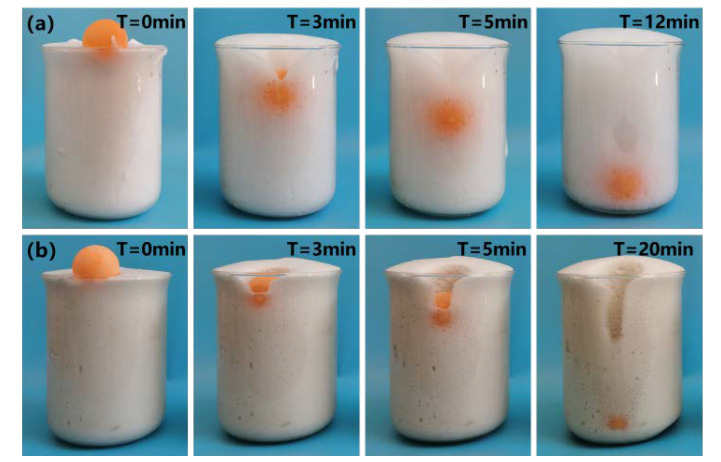
Foam stability test chart: (**a**) SF; (**b**) AF.

**Figure 5:**
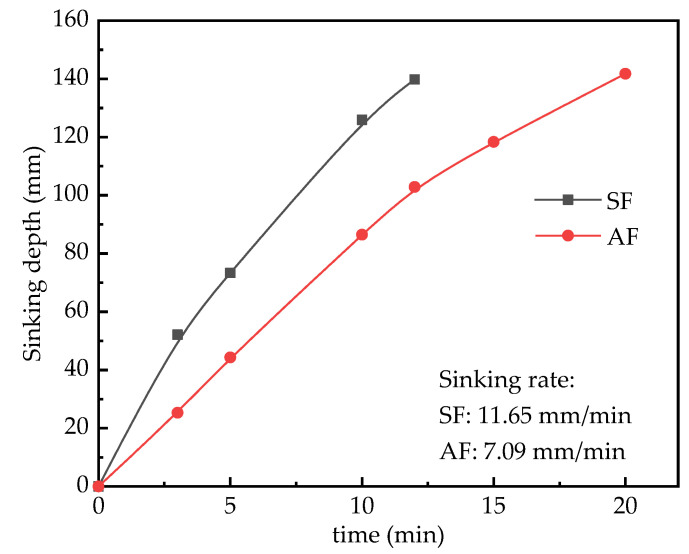
The settlement curve.

Figure 4 shows the position of plastic spheres in the SF and AF foams at different moments. Figure 5 shows the sink depth curve of the spheres, which reflects the sink depth of the spheres as a function of time.

From Figure 4, it can be seen that AF and SF with similar conventional performance indicators showed significant differences in this test. The plastic ball in the SF-type foam sank to one-third from the top of the cup after 3 min, while the plastic ball in the AF-type foam was just submerged in the foam, and the final time for the ball in the SF-type and AF-type foams to reach the bottom of the cup was 12 min and 20 min, respectively. The sinking curves of the plastic ball shown in Figure 5 also show that the sinking rates of the spheres in AF-type and SF-type foams were 7.09 mm/min and 11.65 mm/min, respectively. It is clear that the sinking rate of the plastic ball in AF-type foam was lower than that in SF-type foam, which indicates that the AF-type foam is more resistant to ball sinking than the SF-type foam. This result also indicates that the AF foam has higher stability than SF foams. The possible reasons for the better performance of AF-type foam are as follows: the AF-type foaming agent is a protein-based foaming agent, and the essence of its foaming is the degradation of the protein in the foaming agent. In the preparation of foam via physical foaming methods, the peptide bonds of protein macromolecules break under the action of high-pressure air, forming many small molecules with hydrophobic groups. These hydrophobic groups may have the effect of reducing the surface tension of the solution, and the strong hydrogen bonding between the molecular groups can help to form a mechanically stronger foam liquid membrane, which makes the foam wall tough and stable [15]. Therefore, the foam prepared by AF-type foaming agents has better performance.

### 3.2. Microanalysis

#### 3.2.1. Pore Structure

**Figure 6:**
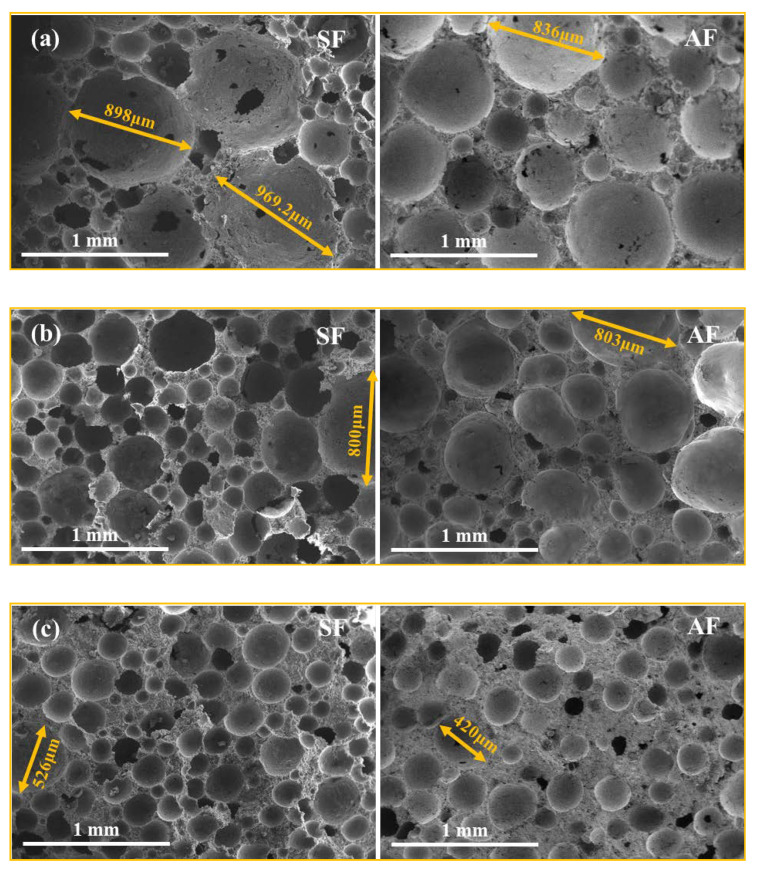

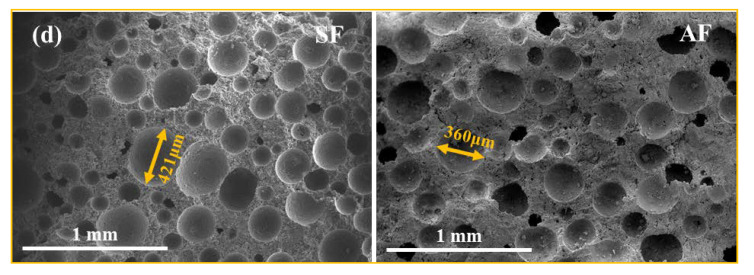
SEM of internal pores of FMLSB: (**a**) 437.5 kg/m^3^, (**b**) 670 kg/m^3^, (**c**) 902.5 kg/m^3^, (**d**) 1170 kg/m^3^.

Figure 6 shows the SEM images of the pore structure of the FMLSB prepared with SF-type and AF-type foaming agents (hereafter referred to as SF-FMLSB and AF-FMLSB, respectively). It can be observed that the pore diameters of both SF-FMLSB and AF-FMLSB gradually decreased as the wet density increased. The reason for this was the high foam content of low-wet-density FMLSB (437.5 kg/m^3^ and 670 kg/m^3^) and the low content of cementitious material, which was insufficient to wrap the bubbles, resulting in the continuous expansion of bubbles before the FMLSB hardened. In contrast, the high-wet-density FMLSB (902.5 kg/m^3^ and 1170 kg/m^3^) has a high content of cementitious material, which can limit the expansion behavior of the pores. The diameter of the largest pores in the observed area of SF-FMLSB and AF-FMLSB at the four wet densities was 969 μm, 800 μm, 526 μm, and 421 μm; and 836 μm, 803 μm, 420 μm, and 360 μm, respectively. Obviously, not only is the maximum pore diameter of AF-FMLSB smaller than that of SF-FMLSB, the pores of AF-FMLSB are also less connected. This is also evidence of the better stability of AF foam.

**Figure 7:**
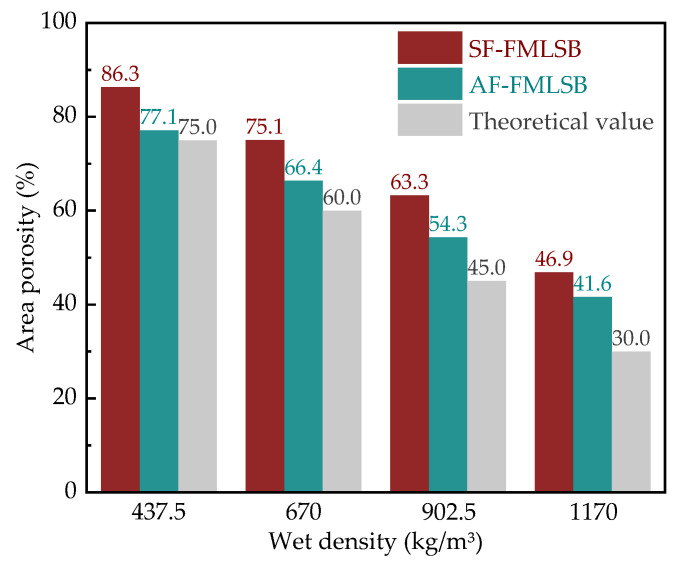
Area porosity of SF-type and AF-type FMLSB.

Figure 7 shows the area porosity of SF-FMLSB and AF-FMLSB obtained by IPP calculation after binarization (where the theoretical value is the ratio of the volume of foam mixed with a cubic volume according to the mix proportion). It can be seen that the area porosity of both SF-FMLSB and AF-FMLSB is different from the theoretical value due to different degrees of expansion of the bubbles. Corresponding to four wet densities (437.5 kg/m^3^, 670 kg/m^3^, 902.5 kg/m^3^, and 1170 kg/m^3^), the area porosity of SF-FMLSB was 86.3%, 75.0%, 63.3%, and 46.9%, respectively, which obviously had a large gap with the theoretical value. The area porosity of AF-FMLSB was closer to the theoretical values, which at 77.1%, 66.4%, 54.3%, and 41.6%, respectively. This result indicates that the SF foam is less stable, and the corresponding SF-FMLSB bubbles’ expansion phenomenon is obvious. AF-FMLSB bubbles’ expansion was much lower, due to the high strength of the AF foam film and the toughness and stability of the bubbles’ walls. It is expected that the FMLSB prepared with an AF-type foaming agent has better mechanical properties.

#### 3.2.2. Skeletal Structure

**Figure 8:**
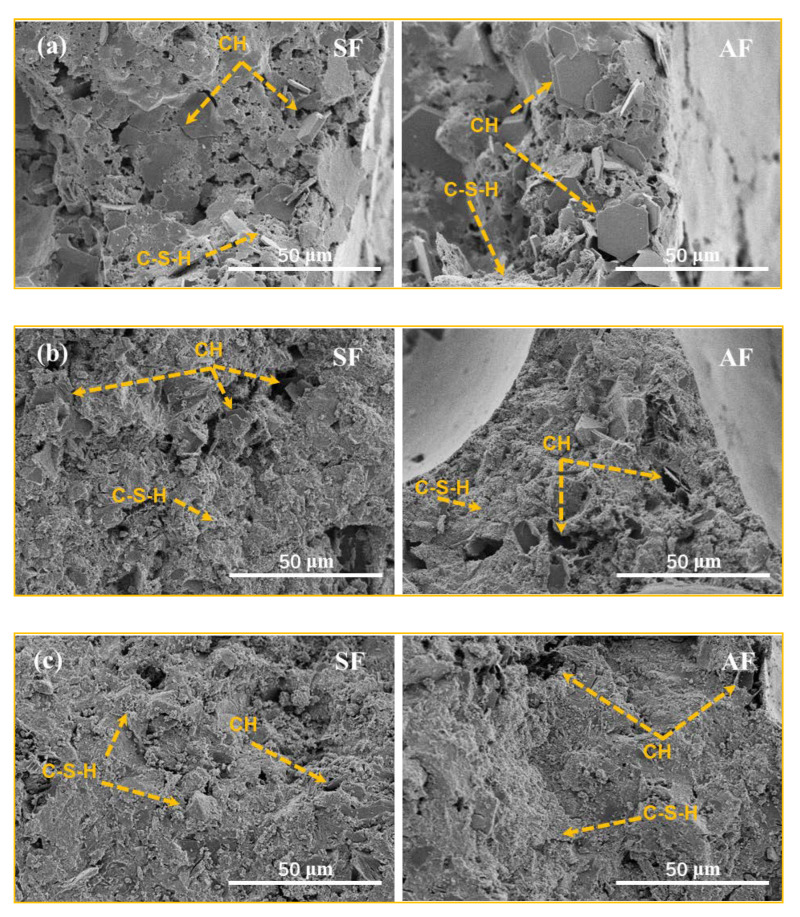

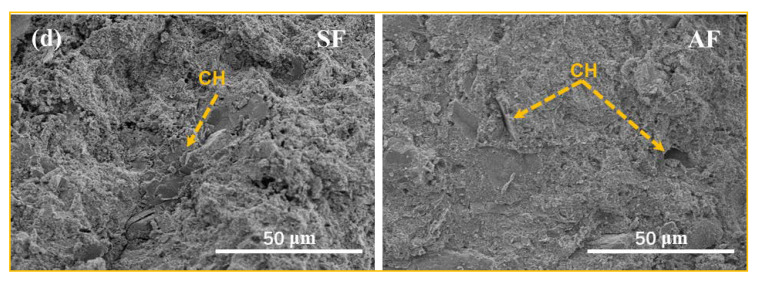
SEM of the skeletal structure of FMLSB: (**a**) 437.5 kg/m^3^, (**b**) 670 kg/m^3^, (**c**) 902.5 kg/m^3^, (**d**) 1170 kg/m^3^.

Figure 8 shows the 1000× SEM images of the skeletal structures of SF-FMLSB and AF-FMLSB at four wet densities. It can be seen that the skeletal structure of SF-FMLSB and AF-FMLSB became denser as the wet density increased. When the wet density was 437.5 kg/m^3^, a large amount of hexagonal lamellar calcium hydroxide (CH) existed in both the SF-FMLSB and AF-FMLSB skeletal structures. These lamellar CH not only have low strength, but also cannot accumulate closely, resulting in the increase in pore space in their skeletal structures. With increasing wet density, the content of gel-like calcium silicate hydrate (C-S-H) in the skeleton increased significantly and filled the pores between CH. Finally, at wet density of 902.5 kg/m^3^ and 1170 kg/m^3^, there were no longer obvious visible hexagonal lamellar CH in the skeletal structures of SF-FMLSB and AF-FMLSB, and a large amount of amorphous C-S-H connected CH to the whole. The BTs also played a microfilling role in the skeletal structures, and some of the SiO_2_ and Al_2_O_3_ would participate in the hydration reaction to generate calcium aluminate hydrate (C-A-H) and C-S-H [6], forming a dense skeletal structure. By comparing the SEM images of the skeletal structures of SF-FMLSB and AF-FMLSB at the four wet densities, it could also be seen that their hydration products and the degree of hydration were basically equivalent. It can be concluded that SF-type and AF-type foaming agents have no effect on the degree of hydration or the hardening of the FMLSB.

### 3.3. Macro Performance Analysis

#### 3.3.1. Uniaxial Compressive Performance

**Figure 9:**
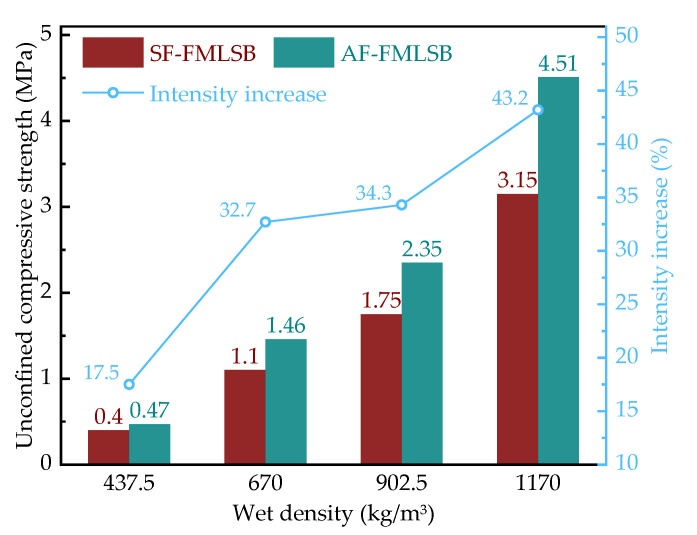
28 d UCS of SF-FMLSB and AF-FMLSB.

The 28 d UCS of SF-FMLSB and AF-FMLSB for the four wet densities is shown in Figure 9. It is shown that the UCS of both SF-FMLSB and AF-FMLSB increased with increasing wet density. This is because the higher the wet density, the less foam content in the FMLSB, and the stress concentration phenomenon gradually diminishes [28]. In addition, at the same wet density level, the UCS of AF-FMLSB is significantly higher than that of SF-FMLSB, and the higher the wet density the greater the increase. For example, at a wet density of 437.5 kg/m^3^, the UCS of AF-FMLSB is 17.5% higher than that of SF-FMLSB, but at a wet density of 1170 kg/m^3^, the increase reaches 43.2%. The UCS of FMLSB mainly depends on the degree of hardening of the cementitious material and the quality of the internal pores. The microscopic skeletal structure analysis showed that the degree of hardening of the cementitious materials in SF-FMLSB and AF-FMLSB is effectively the same, so the quality of the pores is the key factor affecting the UCS. According to the microscopic pore structure analysis of FMLSB, it can be seen that the area porosity of AF-FMLSB is lower than that of SF-FMLSB, and the stress concentration phenomenon caused by the pores is less, so its mechanical properties are also better.

**Figure 10:**
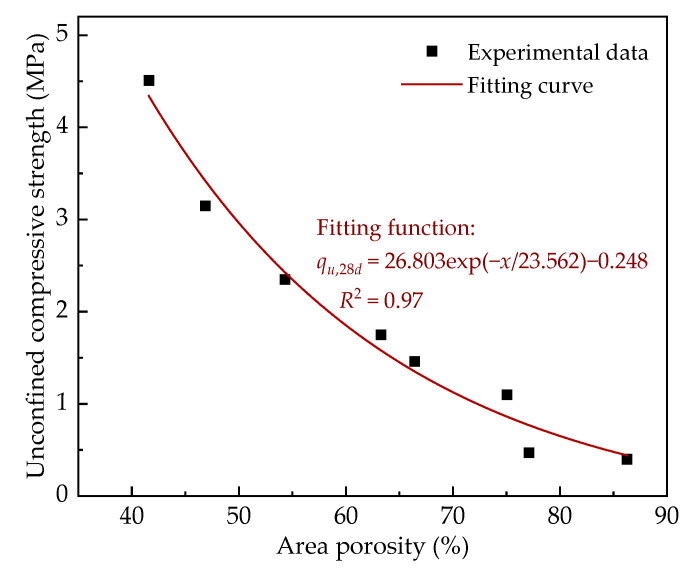
Fitting of experimental data of area porosity and unconfined compressive strength.

Fitting the data of area porosity and UCS of SF-FMLSB and AF-FMLSB, a plot of area porosity versus unconfined compressive strength can be obtained, as shown in Figure 10. It can be seen that the 28 d UCS of FMLSB decreases in an exponential trend as the area porosity increases. The fitting results showed that the correlation coefficient *R*^2^ between the area porosity and unconfined compressive strength of FMLSB reached 0.97, indicating a high fitting correlation. This also confirms that the area porosity is a key factor affecting the mechanical properties of FMLSB. Therefore, it can be considered that the effects of the two foaming agents on the mechanical properties of FMLSB mainly originate from the effects on the area porosity.

#### 3.3.2. Flowability

**Figure 11:**
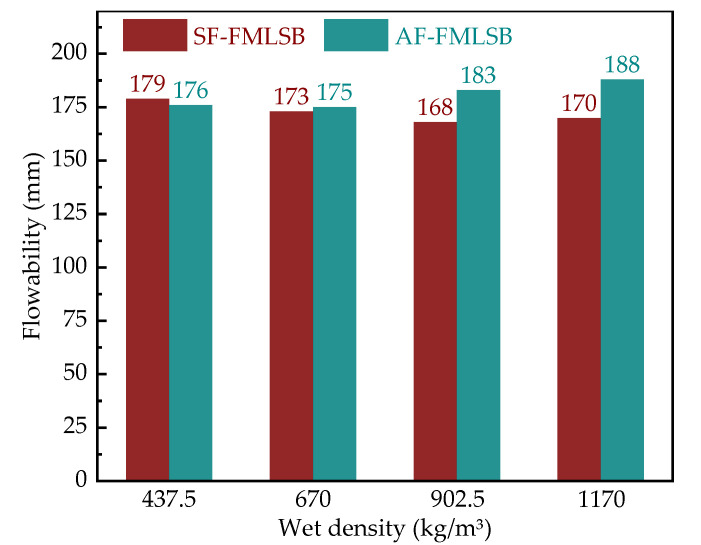
Fluidity of SF-FMLSB and AF-FMLSB.

The results of SF-FMLSB and AF-FMLSB fluidity tests are shown in Figure 11. It can be seen that the fluidity of both was maintained within the range of 160~200 mm, which is in accordance with the requirements of the Chinese Standard CJJ/T 177–2012 [25]. 

In addition, it can also be seen from Figure 11 that the fluidity of SF-FMLSB decreased with the increase in wet density. The reason for this may be related to the BTs’ strong water absorption ability. As the wet density increased from 437.5 kg/m^3^ to 1170 kg/m^3^, the BT content in FMLSB increased from 20% to 28.6%. With the same water–cement ratio, the free water content in FMLSB with low BT content is higher and, therefore, it has higher fluidity. Unlike SF-FMLSB, the fluidity of AF-FMLSB increased with the increase in wet density. This may have been due to the presence of more hydrophobic groups in the foam prepared with the AF-type foaming agent, which play the role of a water-reducing agent. In the foam stability test, it was also discovered that the viscous resistance between AF-type foam particles is lower than that of SF-type particles. This makes the bubbles easier to separate from one another under the self-weight of the FMLSB slurry. Therefore, when the wet density increases, the self-weight of the FMLSB slurry increases, and the fluidity increases accordingly; this factor has a greater impact on the fluidity than that of the BTs. In contrast, SF foams have a high viscous resistance and are not easily separated under the self-weight of the FMLSB slurry, resulting in a lower fluidity of SF-FMLSB than that of AF-FMLSB at higher wet densities.

It is worth noting that there are no significant differences in fluidity between SF-FMLSB and AF-FMLSB at low density. The reason for this is the high content of low-density FMLSB foam, which leads to an increase in the discontinuity of the mixture. At this time, the viscous resistance between the bubbles increases due to them touching one another [29] which, in turn, leads to a decrease in fluidity. This also indicates that at low wet density, the foam content is the main factor affecting the fluidity of FMLSB, rather than the type of foaming agent.

#### 3.3.3. Volume of Water Absorption

**Figure 12:**
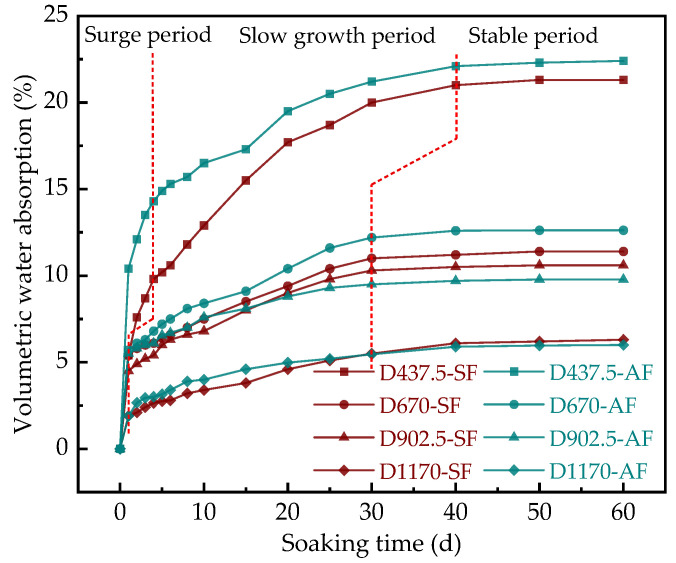
The variation in the volume of water absorption by FMLSB with time.

The curves of the volume of water absorption by SF-FMLSB and AF-FMLSB with soaking time are shown in Figure 12. According to Figure 12, the volume of water absorption by SF-FMLSB and AF-FMLSB decreases with the increase in wet density, and the change process can be divided into three stages: surge period, slow growth period, and stable period. 

It can be seen from Figure 12 that at low wet density (437.5 kg/m^3^ and 670 kg/m^3^), the volume of water absorption by AF-FMLSB at each stage was greater than that by SF-FMLSB. The volume of water absorption by low-wet-density SF-FMLSB and AF-FMLSB reached the equilibrium after soaking for 60 days; the stable values were 21.3%, 11.4% and 22.4%, 12.6%, respectively. It is worth noting that when the wet density was 437.5 kg/m^3^, the growth rate of the volume of water absorption by AF-FMLSB during the surge period was significantly greater than that of SF-FMLSB, which may have been related to the more open pores on its surface. Furthermore, the situation was different at high wet density (902.5 kg/m^3^ and 1170 kg/m^3^). The volume of water absorption by AF-FMLSB increased at a higher rate during the surge period, but at a lower rate than that of SF-FMLSB during the slow growth period, and the volume of water absorption by AF-FMLSB started to be lower than that by SF-FMLSB at that stage. At the end of soaking, the stable values of SF-FMLSB and AF-FMLSB at two wet densities were 10.6%, 6.3% and 9.8%, 6.0%, respectively. In general, in order to have good water resistance, FMLSB with low wet density is more suitable for the use of SF as a foaming agent, while FMLSB with high wet density is suitable for the use of AF as a foaming agent.

## 4. Conclusions

In order to understand the influence of the foaming agent on the performance of FMLSB, the macroscopic physical and mechanical properties, along with the microscopic porosity and skeletal structure characteristics of FMLSB with AF and SF foaming agents, were investigated in this paper. The following conclusions can be drawn:

The foam stability test and microscopic analysis results show that the stability of AF foam is stronger than that of SF foam due to the strong hydrogen bonding between the molecular groups of AF foam. This also helps to reduce the expansion of AF foam in the fresh mixture, thereby reducing the porosity of the hardened AF-FMLSB. Furthermore, the kind of foaming agent has no significant effect on the degree of hydration and hardening of FMLSB.

The 28 d unconfined compressive strength of FMLSB tends to decrease exponentially with the increase in area porosity in the four wet density ranges. Due to the lower area porosity of AF-FMLSB, its compressive strength is significantly higher than that of SF-FMLSB, with an enhancement of up to 43.2%. This indicates that the difference in area porosity of FMLSB caused by different foaming agents is a key factor affecting the mechanical properties of FMLSB. 

The fluidity of AF-FMLSB and SF-FMLSB has different patterns of variation with wet density. At low density, the foam volume fraction is the main factor affecting the fluidity, and the difference in fluidity between AF-FMLSB and SF-FMLSB is not significant, but AF-FMLSB’s fluidity is greater at high density than that of SF-FMLSB, due to the presence of hydrophobic groups in AF foam that act as water-reducing agents.

The volume of water absorption rate by FMLSB can be divided into three stages: surge period, slow growth period, and stable period. At low wet density, the water absorption of AF-FMLSB was greater than that of SF-FMLSB at all soaking stages, but the opposite was true at high wet density, indicating that the type of foaming agent also has an effect on the volumetric water absorption.

In the engineering applications of FMLSB, the foaming agent should be selected according to the wet density, fluidity, compressive strength, and volumetric water absorption of the project. For example, for some filling projects in karst areas with low strength requirements, the design value of the wet density of FMLSB is usually not high. Therefore, choosing SF as a foaming agent can lead to better water resistance. 

## Figures and Tables

**Table 1:** Physical parameters and mineral composition of BTs.

Specific Gravity of Solid Particles	Liquid Limit (%)	Plastic Limit (%)	Bulk Density (g/cm^3^)	Void Ratio	Chemical Composition/%					
					Al_2_O_3_	SiO_2_	Fe_2_O_3_	CaO	TiO_2_	K_2_O
2.76	70.43	31.96	1.58	0.96	38.10	28.32	14.93	0.32	1.70	0.78

**Table 2:** Performance indices of SF and AF.

Foaming Agent Type	pH Value	Dilution Ratios	Foam Multiple	Water Secretion (mL/h)	Sink Distance (mm/h)
SF	7.5	1:40	>40	16	< 10
AF	7.0	1:30	>40	12	< 10

**Table 3:** Chemical composition and mineral composition of BTs.

Designed Wet Density	Cement (kg/m^3^)	BTs (kg/m^3^)	Foam Content (L/m^3^)	Water/Solid Ratio
D437.5	200	50	750	0.6
D670	300	100	600	0.6
D902.5	400	150	450	0.6
D1170	500	200	300	0.6

## Data Availability

Some or all data, models, and code that support the findings of this study are available from the corresponding author upon reasonable request.
